# Factors Influencing the Oxygen Isotopes of Grape Berry Water: Some Remarks

**DOI:** 10.1155/ijfo/8946352

**Published:** 2026-01-05

**Authors:** Mattia Rossi, Tiziano Boschetti, Enricomaria Selmo, Francesco Caraffini, Sofia Ramigni, Paola Iacumin

**Affiliations:** ^1^ Department of Chemical, Life and Environmental Sustainability Sciences, University of Parma, Parma, Italy, unipr.it

## Abstract

Several chemical and isotopic methods have been suggested for tracing the wine origin in relation to the climate/meteorological conditions of the vineyard areas. IRMS (Isotope Ratio Mass Spectrometer) is one of these tools. In the Oltrepò Pavese (Lombardia Region, Pavia Province, Italy) and in the Illasi/Mezzane areas (Veneto Region, Verona Province, Italy), for the harvests of 2021 and 2022, a linear correlation between *δ* ^18^O of the grape must water (*δ* ^18^O_m_) and the potential evapotranspiration (ET_O_) and crop evapotranspiration (ET_C_) has been found. ET_O_ and ET_C_ were calculated according to the FAO‐56 model using meteorological data from the meteorological stations located close to the vineyards studied. We can state that the intercept obtained for the linear correlation *δ* ^18^O_m_ on ET_C_ is an estimate of the isotopic features of the water absorbed by plants during the late stage of the grape ripening. The obtained data allowed us to estimate the isotopic enrichment factor *ε* ^18^O_m−PW_ (m = grape must water and PW = precipitation water), which depends on climatic/meteorological conditions. Subsequently, the climatic parameters that mostly influence the oxygen isotope variation in grape must water have been identified using a correlation coefficient.

## 1. Introduction

The production of the wine is one of the biggest activities done in the world. In 2024, the world wine production reached about 225.8 MhL; 61.2% of the production is from Europe, notably Italy (44.1 MhL), France (36.1 MhL) and Spain (31.0 MhL) [1]. With the aim of protecting the national identity, the European Union established several legislative procedures and many Regulations. Regulation (EC) No. 479/2008, integrated with the Single CMO (Reg. (EC) No. 1234/2009), governs the rules about the common market organisation (CMO) in the common agricultural policy (CAP), including also the rules concerning the origin of the wine. This is because the chemical features of the wine are strongly dependent on climate and yearly influenced by the meteorological conditions.

Many chemical and isotopic methods have been proposed to trace the origin of the wine and to have indication on climate/meteorological conditions of the areas of the vineyards. The isotope ratio mass spectrometry (IRMS) is one of the largely used method. By this method, it is possible to determine the ratio between different isotopes of the same element, in particular H, C, N and O. To compare the different values of the isotope ratio, the parameter delta (*δ*) is used [2]:

(1) δi/jERM=E i/E jsE i/E jRM−1∙103‰,

where E i/E js and E i/E jRM are the ratios between the abundance of the less abundant isotope  ^i^E to the most abundant isotope  ^j^E present in the substance s of interest and in a reference material RM, and ‰ = 10^−3^ . In the OIV method [3]  ^i^E and  ^j^E are, respectively,  ^18^O and ^16^O of the water must molecules, and RM is VSMOW, the primary international reference material commonly used for oxygen and hydrogen isotope in the water molecule.

The *δ* ^18^O value of water of grape berries reflects the climate/meteorological conditions of the areas of the vineyard before the harvest because, during the ripening of grapes, berries undergo a transpiration process [4]. Different studies have been done to connect the climate variables to the isotopic composition of the different compounds of wines [5–9]. Ingraham and Caldwell [5] studied how crop evapotranspiration modifies the water *δ* ^18^O value of wines. On the other hand, Camin et al. [8] studied how the climate and geographical variables influence the water *δ* ^18^O value of wines, and the *δ* ^2^H and *δ* ^13^C values of the wine ethanol.

Furthermore, it is well known that isotope fractionation does not occur between the soil water and water adsorbed by plant roots; actually, it has been seen that the isotope composition of xylem water has the same composition as the water adsorbed [10–13]. Unfortunately, in this study it was not possible to carry out analysis for the composition of xylem water.

In this paper, we will define:

i. The best linear correlation between both potential evapotranspiration and crop evapotranspiration values, calculated according to the FAO‐56 model [14] and the *δ* ^18^O values of the must water:
ii. The significance of the intercept in the regression line *δ* ^18^O values of the must water on the evapotranspiration.
iii. The role of the climatic variables (air temperature, wind speed, solar radiation and relative humidity) which mostly influence *δ* ^18^O of the must water.

## 2. Material and Methods

### 2.1. Areas of Investigation

**Figure 1:**
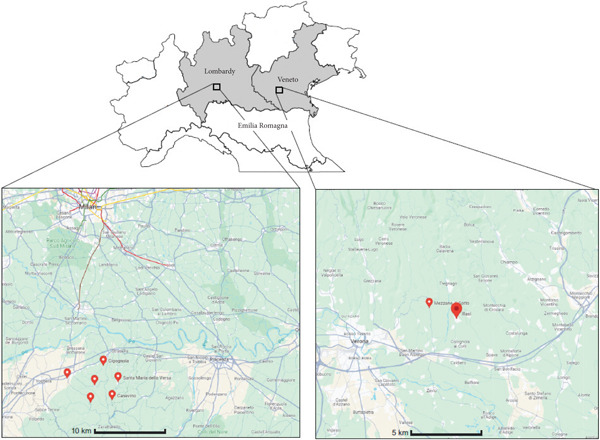
Sampling points in the different areas for the harvest of 2021 and 2022.

To achieve the goals listed above, we identified two different areas in Northern Italy: Oltrepò Pavese (Pavia province, Lombardia Region, Italy) and Illasi/Mezzane area (Verona province, Veneto region, Italy) (Figure 1). In Oltrepò Pavese, six areas—Santa Maria della Versa, Cigognola, Canevino, Montalto Pavese, Montebello della Battaglia and Borgoratto Mormorolo—were considered. For each area, the number of selected vineyards was variable: at Borgoratto Mormorolo, Canevino, Santa Maria della Versa and Cigognola, one vineyard for each locality; at Montebello della Battaglia two vineyards; and, finally, at Montalto Pavese, three different vineyards. Each vineyard was equipped with a meteorological station. For Illasi/Mezzane, nine and eight vineyards in 2021 and 2022, respectively, were considered. Meteorological data were furnished by ARPAV (Regional Agency for the Environment Protection of Veneto) for the Illasi/Mezzane area, whereas for the Oltrepò Pavese area, a meteorological station was located in each vineyard considered.

The climate of the areas of Borgoratto Mormorolo, Montalto Pavese, Santa Maria della Versa, Montebello della Battaglia, Cigognola and Illasi/Mezzane is classified as humid subtropical (Cfa, according to the Köppen–Geiger Climate classification), a temperate climate with hot and humid summers and cool to mild winters [15]. The area of Canevino is characterized by temperate oceanic climate (Cfb according to the Köppen–Geiger Climate classification) with cool to warm summers and cool to mild winters. Vineyards located in the Oltrepò Pavese area are all characterized by the following geological formations: “Val Lauretta” (Eocene–Paleocene), consisting of a rhythmic succession of limestones, marls and sandstone; “Arenarie di Ranzano” (Upper Oligocene–Upper Eocene), with frequently cemented conglomerates, sandstones/sands and “Complesso indifferenziato” (Oligocene–Middle Eocene?), composed of arenaceous, clay and limestone. For all the formations, X‐ray diffraction analysis indicated the occurrence of calcite, quartz, feldspar and clay minerals. The clay portion (< 2 *μ*m) consists of smectite, illite, chlorite and kaolinite.

At Illasi/Mezzane, the vineyards are located on a predominantly calcareous/dolomitic substrate from Jurassic (Calcari Grigi di Noriglio, Oolitico di san Vigilio, in part dolomitized and Rosso Ammonitico) to Cretaceous (Maiolica and Scaglia Rossa) up to Eocene (Calcari nummulitici). X‐ray diffraction investigation revealed calcite, dolomite, quartz, feldspars, and clay minerals (kaolinite, chlorite, illite and smectite).

### 2.2. Sampling

During the harvest of 2021 and 2022, bunches of grapes were collected during a period of 4 weeks in the Oltrepò Pavese area (Figure 1): one sampling per week at Borgoratto Mormorolo, Canevino, Cigognola and Santa Maria della Versa; two samplings per week at Montebello della Battaglia and, finally, three samplings per week at Montalto Pavese. Vineyards were not irrigated; thus, atmospheric water is the only source of water for the area. Grapes were collected at the same points in both years. Furthermore, in 2022 the monthly rainfall was sampled in two different villages: Santa Maria della Versa and Cigognola.

At Illasi/Mezzane (Figure 1), eight bunch of grapes were collected in 2021 and nine in 2022.

### 2.3. Analytical Methods

**Figure 2:**
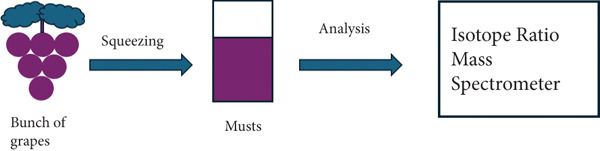
Scheme from sample collection to sample analysis.

To obtain the must, each bunch of grapes was squeezed right after collection. After squeezing, the samples were filtered with 0.45‐*μ*m filters to eliminate the solid material (peels and seeds) (Figure 2).

According to the OIV method [3], must samples as well as the reference materials were analysed without any pretreatment. A total of 4 cm^3^ of must samples were equilibrated at (18 ± 0.1)°C with a small amount of pure CO_2_ using a HDO equilibrator (ThermoFinnigan, Bremen, Germany). The *δ* ^18^O values of CO_2_ equilibrated with water were determined with a Delta Plus isotope ratio mass spectrometer (ThermoFinnigan) in line with the equilibrator. The samples were stirred during the time of equilibration. Two different laboratory standards (very low salinity waters) with *δ* ^18^O values of (−8.69 ± 0.05, standard error) ‰ and (−0.02 ± 0.02) ‰, calibrated against the international standards VSMOW2 and SLAP2, were used to normalise the raw data. The prediction uncertainty [16, 17] on the sample *δ* ^18^O value was 0.08‰ for meteoric water samples and 0.11‰ for the must water samples (*α* = 0.05).

## 3. Results and Discussion

### 3.1. Relationship Between *δ* ^18^O and Daily Potential Evapotranspiration (ET_O_)

Thanks to the meteorological station near the vineyards investigated, the daily potential evapotranspiration ET_O_ [14] for the six sites of Oltrepò Pavese and for the sites of Illasi/Mezzane was estimated. The FAO‐56 formula was used:

(2) ETO=0.408900273∙Δ∙Rn−G+γ∙ T+∙u2∙es−eaΔ+γ∙10.34+∙u2,

where R_n_ is the net radiation at the crop surface [MJ m^−2^ day^−1^], G is the soil heat flux density [MJ m^−2^ day^−1^], e_a_ is the actual water vapour pressure [kPa], e_s_ is the saturation vapour pressure [kPa], T is the mean daily air temperature [°C] at 2 m from the soil surface, *Δ* is the slope vapour pressure curve [kPa°C^−1^], *γ* is the psychrometric constant [kPa°C^−1^] and u_2_ is the wind speed [m s^−1^] at 2 m from the soil. The potential evapotranspiration represents the evaporating power of the atmosphere at a specific location and time of the year and does not consider the crop characteristics and soil factors. As a result, potential evapotranspiration values depend only on climate variables (solar radiation, relative humidity, air temperature and wind speed)

Hereafter, we assume that the isotopic value *δ* ^18^O_m_ of the grape must water is dependent on evapotranspiration [5, 18]. A script in Matlab (Matlab, 2020; Version 2020a; Natick, Massachusetts: The MathWorks Inc.) was done to automate the calculation of the daily evapotranspiration for each site in the different years studied. This programme was validated using the daily climatic data of the literature (Example 18, Chapter 4 of [14]). As discussed above, the isotopic features of oxygen are dependent on the meteorological conditions preceding the harvest. The evapotranspiration is a parameter which tries of summarising these conditions. Thus, a correlation between the average $\bar{\text{ET}}$_O_ value, calculated for n days before the harvest, and *δ* ^18^O_m_ is expected.

**Figure 3:**
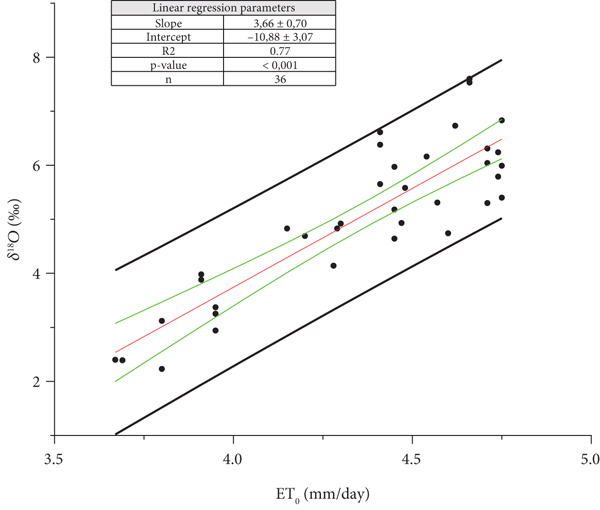
Ordinary least square (OLS) regression between the mean of daily potential evapotranspiration ($\bar{ET}$_O_) for the 44 days preceding the day of each harvest, and *δ* ^18^O_m_ of the must water for the Year 2021 (*p* value < 0.001) from Oltrepò Pavese. The back straight lines refer to the prediction bands, the green ones to the confidence bands. The OLS regression line is reported in red.

**Figure 4:**
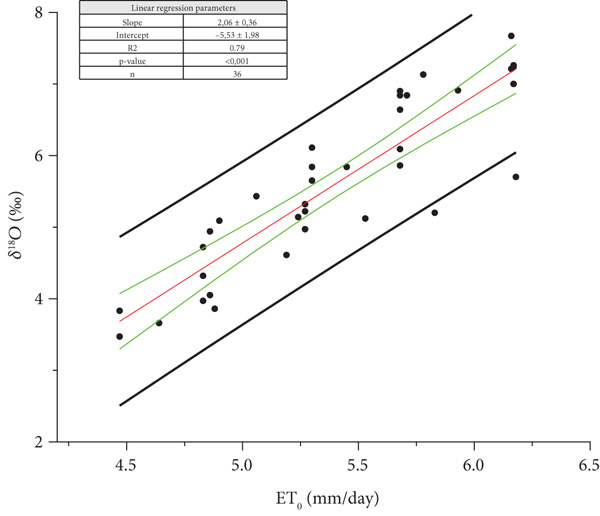
Ordinary least square (OLS) regression between the mean of daily potential evapotranspiration ($\bar{ET}$_O_) for the 58 days preceding the day of each harvest, and *δ* ^18^O_m_ of the must water for the Year 2022 (*p* value < 0.001) from Oltrepò Pavese. The back straight lines refer to the prediction bands, the green ones to the confidence bands. The OLS regression line is reported in red.

For 2021, in the Oltrepò Pavese, the average, $\bar{\text{ET}}$_O_, of the values obtained for the 44 days preceding each harvest day gave the best linear correlation with *δ* ^18^O_m_ of each harvest (Figure 3). For 2022, the best linear fitting was found considering the average value, $\bar{\text{ET}}$_O_, for 58 days (Figure 4).

For both the years, *δ* ^18^O_m_ is strongly related to$\;\bar{\text{ET}}$_O_. In 2022, a very warm year in Europe [19], $\bar{\text{ET}}$_O_ was higher than in 2021. Indeed, in our areas, the harvest began 2 weeks before than in 2021. In both Figures 3 and 4, the intercept (*δ* ^18^O for$\;\bar{\text{ET}}$_O_ = 0) of the straight line $\bar{\text{ET}}$_O_ on *δ* ^18^O_m_ is negative and represents the estimate of the delta value of plant water (*δ* ^18^O_pW_) before the isotopic enrichment due to the transpiration.

The same research was carried out for the harvest 2021 and 2022 in the Illasi/Mezzane site. We found a good linear relationship between $\bar{\text{E}{\text{T}}_{\text{O}}}$, and *δ* ^18^O_m_, but due to the low samples number (*n* = 8 and *n* = 9 for 2021 and 2022, respectively) the linear equations obtained did not give a good statistical information.

### 3.2. Relationship Between *δ* ^18^O and Daily Crop Evapotranspiration (ET_C_)

Crop evapotranspiration under standard conditions, denoted as ET_C_, is the evapotranspiration from disease‐free, well‐fertilized crops, grown in large fields under optimum soil water conditions and achieving full production under the given climatic conditions. ET_C_ is obtained by multiplying ET_O_ by K_C_ [20]:

(3) ETC=KC ET0.

The “crop coefficient”, K_C_, reflects the effect of the characteristics that distinguish a typical field crop from the grass of reference. Consequently, different crops have different K_C_ coefficients. Furthermore, the value of K_C_ depends on (i) four growth stages: initial, crop development, mid season and late season; (ii) the stage length (in days), which depends on the latitude; (iii) type of crop; (iv) height of the crop (h_P_).

Ingraham et al. [5] and Caldwell [18] found a good linear relationship between the crop evapotranspiration (ET_C_) of the month before the harvest and the value of *δ* ^18^O of the musts/wine water (*R* ^2^ = 0.93). In addition, Caldwell [18] indicated that the intersection point between the meteoric water line (MWL) of the local precipitation and the transpiration line could indicate the isotopic composition of the water absorbed by roots. This is possible because the adsorption of the water by the roots does not generate isotope fractionation [10, 21–28]. Using a script in Matlab, we tried to verify if also in our areas there was a significant linear correlation between the mean daily crop evapotranspiration of the days before the harvest and *δ* ^18^O_m_ of the must waters, and if using ET_C_ in place of ET_O_ the correlation improves.

#### 3.2.1. Oltrepò Pavese

In this work, the crop is grape of vineyards located at middle latitude. The K_C(initial season)_, K_C(middle season)_ and K_C(end season)_ values are tabulated (0.30, 0.70, and 0.45, respectively). The meteorological conditions for which the tabulated values were obtained are the following: minimum daily relative humidity (h_min_) = 45% and wind speed = 2 m/s (u_2_) (measured at 2 m above the ground). If h_min_ and/or u_2_ is/are different from the values reported above, the K_C_ values must be adjusted using the following equation [14]:

(4) KCadjusted=KCtabulated+0.040.004 u2−2−hmin−45∙hp30.3.

**Figure 5:**
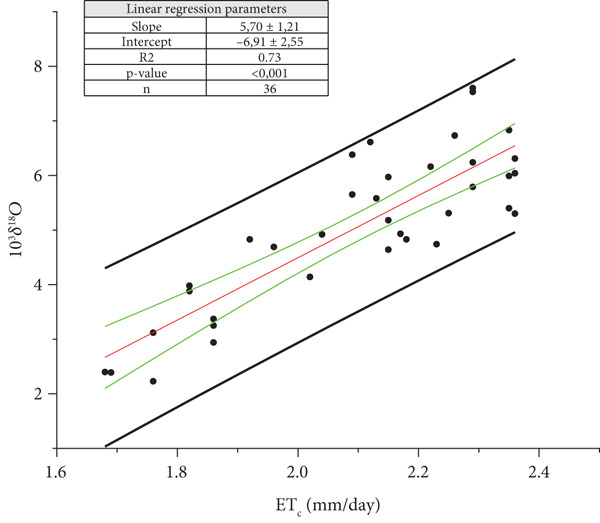
Ordinary least square (OLS) regression between the means of daily crop evapotranspiration ($\bar{ETc}$) and *δ* ^18^O_m_ of the must water for the Year 2021 (*p* value < 0.001). The back straight lines refer to the prediction bands and the green ones to the confidence bands. The OLS regression line is reported in red.

**Figure 6:**
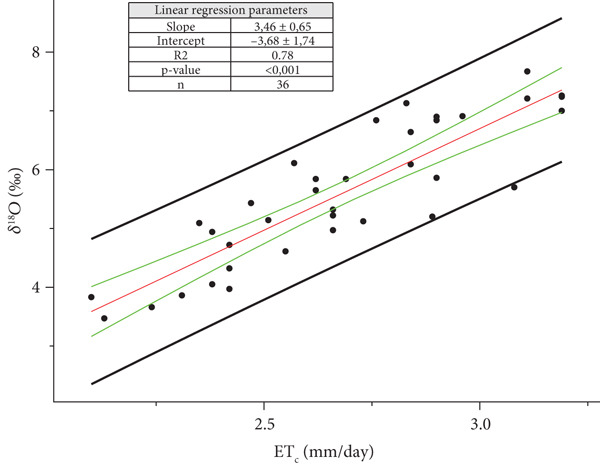
Ordinary least square (OLS) regression between the means of daily crop evapotranspiration $\bar{ETc}$ and *δ* ^18^O_m_ of the must water for the Year 2022 (*p* value < 0.001). The back straight lines refer to the prediction bands and the green ones to the confidence bands. The OLS regression line is reported in red.

The period of our interest is between the middle season and the end season (late season), a period in which the maturity starts. The late season may reach 80 days [14]. Unlike K_C(intial season)_, K_C(middle season),_ and K_C(end season)_ values, the K_C(late season)_ value is the result of an interpolation between K_C(middle season)_ and K_C(end season)_. We tried to estimate K_C(late season)_ using Equation (4), starting from the K_C(end season)_ value and replacing the “standard” values of h_min_ and u_2_ with real daily values. If the obtained values were within 0.45 and 0.7, they were used, whereas values lower than 0.45 were rejected and replaced with tabulated values. For example, if the result was 0.55, we kept this value; if the value was 0.40, we kept the tabulated value, that is, 0.45. In this way, we tried to “draw” the line of late season described in Allen et al. (Figure 34 in [20]). After that, the linear correlation between mean $\bar{\text{ET}}$_C_ and *δ* ^18^O_m_ was verified. For 2021, we obtained the best relationship using a value of $\bar{\text{ET}}$_C_ calculated starting from 41 days preceding each harvest date (Figure 5); for 2022, starting from 58 days before (Figure 6).

In case the value $\bar{\text{ETc}}$ is equal to zero, the *δ* ^18^O is equal to the intercept A of the regression line Y = B X + A. For 2021, is

(5) A2021=δ18OmET¯C=0=δ18OpW 2021=−6.911.26±‰,

and for 2022,

(6) A2021=δ18OmET¯C=0=δ18OpW 2022=−3.680.86±‰,

(uncertainty at significant level *α* = 0.32), where *δ* ^18^O_pW_ indicates the weighted mean value of the water adsorbed by plants starting from 41 (Equation (5)) to 58 (Equation (6)) days before the harvest. Since isotope fractionation does not occur between soil water and water absorbed by plant roots [10], *δ* ^18^O_pW_ estimates the soil value (*δ* ^18^O_sW_).

In 2022, the 58th day preceding each harvest fell in the final 3 days of June, July, August and September (Table 1). At the end of June, rain was totally absent. For this reason, the weighted mean of *δ* ^18^O_MW_ for meteoric water was calculated only for July, August and September. The value for the two sites considered together is the following:

(7) δ18OMW=−4.010.08±‰standard error,

**Table 1:** Monthly precipitation (mm) and 10^3^ *δ* ^18^O_MW_ values for the meteoric water for the sites of Santa Maria della Versa and Cigognola (Year 2022). In May, the precipitation in Santa Maria della Versa was not analysed because of a technical problem. Uncertainty on *δ* ^18^O has been calculated as prediction uncertainty at 95% probability.

**Months 2022**	**mm**	10^3^ **δ** ^18^ **O**_**M** **W**_ (±0.08)
Santa Maria della Versa		
January	13.2	−5.40
February	14.4	−12.81
March	15.4	−8.31
April	22.8	−7.16
May	89.6	n.d.
June	25.2	−1.84
July	126.4	−5.11
August	45.6	−2.57
September	23.2	−5.86
October	5.4	−5.36
November	72.6	−12.76
December	96.2	−13.01
Cigognola		
January	11.4	−5.26
February	13.6	−12.23
March	15.8	−7.37
April	20.6	−6.13
May	70.8	−8.08
June	36	−2.19
July	115.2	−3.70
August	47.6	−2.31
September	18.4	−4.08
October	5.6	−5.85
November	86	−13.23
December	111.2	−14.08

a value which, statistically, cannot be considered significantly different from the intercept of Equation (6) (roughly estimated *p*_same_ value ≈ 0.7 (Student′s t‐test)). This suggests that the source of water for the vine in 2022 is prevalently that of July, August and September. Even if there was a small difference between *δ* ^18^O_MW(2022)_ and *δ* ^18^O_pW (2022)_, it would be so small as to be easily explained by some evaporation of the meteoric water before infiltration and/or in the soil. To be sure that *δ* ^18^O_pW (2022)_ ≈ *δ* ^18^O_MW(2022)_, the xylem water [11–13] should have been extracted.

If intercept represents the mean of soil water taken up by plants during their growth period and the maturity of the fruit, an isotopic enrichment factor, *ε*, between must water and plant water may be calculated. An estimate of the minimum and maximum isotopic enrichment for both 2021 and 2022 are the following:

(8) ε18Om−pW maximum 2021=7.600.11±−−6.911.26±‰1+−6.911.26±‰=14.611.27±‰.

(9) ε18Om−pW minimum 2021=2.230.11±−−6.911.26±‰1+−6.911.26±‰=9.201.27±‰.

(10) ε18Om−pW maximum 2021=7.670.11±−−3.680.86±‰1+−3.680.87±‰=11.390.90±‰.

(11) ε18Om−PW minimum 2022=3.470.11±−−3.680.86±‰1+−3.680.86±‰=7.180.90±‰.

The four different isotopic enrichments depend on the climate/meteorological conditions, because the starting value of *δ* ^18^O_m_ depends on ET_C_. Thus, it is possible to define εO 18m−pW,climate a “climate” isotope enrichment factor, *ε* ^18^O_climate_, depending on air temperature, relative humidity, solar radiation and wind speed. The values *ε* ^18^O_mpW,climate_ is related to nonequilibrium conditions and reflects the climate/meteorological conditions before the harvest. Indeed, the ^18^O enrichment of the must water (due to the preferential evaporation of the lighter water molecules) occurs as diffusion and/or turbulent process, depending on the climate/meteorological conditions.

Table 2 reports the total rain fallen in the 41 days preceding each harvest for 2021 and 58 days for the 2022 year.

**Table 2:** Total rain fallen during the 41 days preceding each harvest for 2021 and 58 days for 2022.

	**Borgoratto Mormorolo**	**Canevino**	**Montalto Pavese**	**Cigognola**	**Montebello della Battaglia**	**Santa Maria della Versa**
Millimetres of rain, 2021						
1st harvest	5.20	5.20	12.60	12.00	38.80	5.00
2d harvest	0.60	0.60	1.00	0.00	0.00	0.00
3d harvest	0.60	3.00	2.20	1.20	1.80	2.40
4th harvest	4.80	39.40	23.00	26.40	22.00	27.60
Millimetres of rain, 2022						
1st harvest	77.20	87.00	90.80	168.40	61.00	171.00
2d harvest	62.80	73.60	77.80	153.60	43.80	158.80
3d harvest	62.80	73.80	78.00	154.00	44.60	158.80
4th harvest	62.80	78.60	82.40	159.80	49.80	162.40

In 2021, in the 41 days preceding the harvests, the rainfall was moderate. Considering that the vineyards were not irrigated manually, we can assume that the water adsorbed by plants came only from water infiltrated in a previous period. The groundwater (GW) sampled in a well at Cigognola gave the following *δ* ^18^O_GWS_ value:

(12) δ18OGW Cigognola 2021=−8.190.08±‰,

a value which is nearly constant during the year. The statistical comparison between the value *δ* ^18^O_GW (Cigognola 2021)_ and the intercept value $\delta^{18}{\text{O}}_{\text{m}\left({\text{ET}}_{\text{C}}=0\right)}=\left(-6.911.26\pm \right)‰$ gives the rough estimate *p*_same value_ ≈ 0.40 (Student′s t‐test). Since in the natural system under consideration, it is expected that the isotope values are not very different from each other (only some delta units), the probability of 0.4 could be not sufficient to guarantee the null hypothesis. In this case, the accepted difference could be easily explained by evaporation both on the atmosphere and in the soil. Moreover, both the best OSL linear regressions, ET_O_ on *δ* ^18^O_GW_ and ET_C_ on *δ* ^18^O_GW_, are related to a shorter period for 2021 (44 and 41 days, respectively) than for 2022 (58 days for both). Table 2 provides an explanation for this difference: in 2022, the rain was abundant before each harvest, whereas in 2021, the rain was much less. Indeed, meteoric water before harvest controls the isotopic enrichment and depletion of grape musts water [5, 18].

The statistical comparison between the two regression lines (OriginPro, Version 2023b. OriginLab Corporation, Northampton, MA, United States) indicates that both intercept and slope are statistically different.

#### 3.2.2. Illasi/Mezzane Site

The same research was carried out for the harvest of 2021 and 2022 in the Illasi‐Mezzane area. However, although a good linear relationship exists between ET_C_ and *δ* ^18^O_m_, its statistical reliability is low because of the low number of samples.

**Figure 7:**
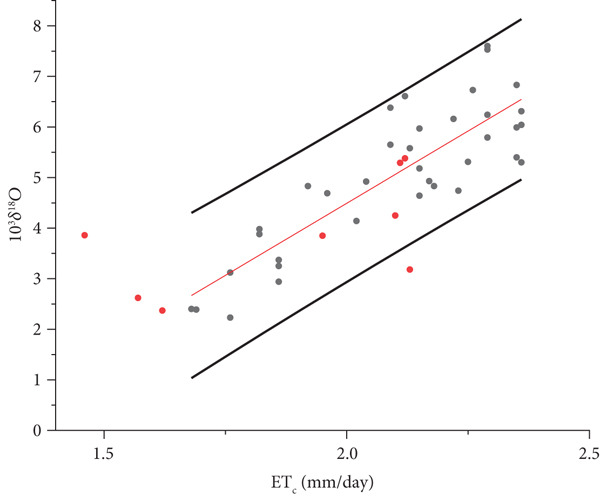
OLS (ordinary least squares) linear correlation between the means of daily crop evapotranspiration $\bar{ETc}$ and *δ* ^18^O_m_ of the must water for the Year 2021 as in Figure 5 with the addition of the Illasi/Mezzane values (red dots).

**Figure 8:**
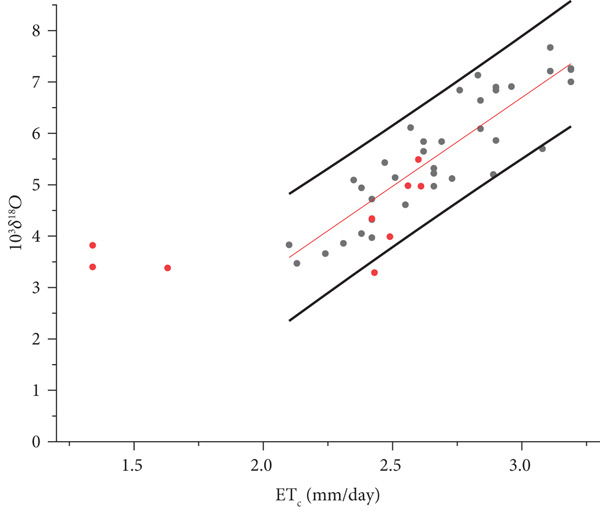
OLS (ordinary least squares) linear correlation between the means of daily crop evapotranspiration $\bar{ETc}$ and *δ* ^18^O_m_ of the must water for the Year 2022 (Figure 6) and Illasi/Mezzane values (red dots).

From Figures 7 and 8, it is evident that some Illasi/Mezzane samples (red points) fall outside the prediction interval of the Oltrepò Pavese regression line. This is due to the different climate conditions characterising the two studied areas. Whereas for the Oltrepò Pavese the end of August marks the beginning of the harvest, which lasts until the end of September, at Illasi‐Mezzane the harvest begins at the end of August and finishes approximately in the middle of October. The samples collected in October have lower values of ET_C_ because solar radiation and temperature decrease during the time.

## 4. Some Considerations on the Meteorological/Climate Parameters

In addition to the good relationship between ET_C_ and *δ* ^18^O_m_, we want to point out what are the meteorological/climate parameters that mostly influence *δ* ^18^O_m_ (Table 3). For this purpose, we have calculated the average of each single meteorological variable already used to calculate the daily ETc values (solar radiation, air temperature, relative humidity and wind speed). The averages are referred to the period (days) for which the best linear relation between ET_C_ and *δ* ^18^O_m_ values (58 days for 2022 and 41 days for 2021 before the harvest) was found.

**Table 3:** Correlation coefficient (*r*) between *δ* ^18^O_m_ values and solar radiation, air temperature, relative humidity and wind speed.

**Relation**	**r**
(*δ* ^18^O, solar radiation)	0.77
(*δ* ^18^O, wind speed)	0.69
(*δ* ^18^O, relative humidity)	−0.47
(*δ* ^18^O, air temperature)	0.61

Solar radiation and air temperature are connected: an increase of solar radiation increases air temperature [29, 30]. As a result, an increase in the temperature of the air increases (i) the evapotranspiration process [31] and (ii) promotes the enrichment in heavy isotope in the grape must water. As wind speed is concerned, a correlation with the evapotranspiration rate exists [32, 33]. During the process of evaporation, a saturated water vapour layer is formed on the liquid layer. This saturated vapour can be replaced with drier air by wind. As a result, an increase in the evaporation rate occurs: the greater the wind speed, the greater the rate of evapotranspiration. The relative humidity has an inverse correlation with the evapotranspiration [32]. This means that the greater the value of relative humidity, the lower the evapotranspiration process.

## 5. Can Be a Predictive Model?

In the introduction, we highlighted how the isotope analysis is one of the most accurate methods to verify the origin and the possible fraud in the wines. After this study, it is fair to ask if the linear model found between *δ* ^18^O_m_ values and ET_C_ can be used to verify the origin and the possible fraud. It is a complex problem, because looking at Figures 5 and 6, it is possible to notice that almost all the data are inside the prediction interval, except for one in Figure 6. If only one data point used to build the linear model is outside, it means that a big error is done. Furthermore, it is also possible to see how large the prediction bands are, highlighting the big dispersion of “predictive” data. All these suggest to us that these linear relations have more descriptive character than a predictive one. For all these reasons, we suggest to each farmer to construct their own *δ* ^18^O_m_ databank each harvest year, according to EU regulation No. 822/97.

## 6. Summary and Conclusions

Isotope and climate data from the Oltrepò Pavese area show that:

- The meteorological conditions can affect the *δ* ^18^O_m_ of grape must water. Good linear relations between both potential evapotranspiration, ET_O_, on *δ* ^18^O_m_ and crop evapotranspiration, ETc, on *δ* ^18^O_m_ exist.
- The intercept of the regression line ET_C_ on *δ* ^18^O_m_ for Oltrepò Pavese gives an estimate of the mean *δ* ^18^O_pW_ value of the water that plants adsorbed during the process of ripening. For 2022, this value is comparable with the weighted mean of precipitation referred to a defined period (58 days) preceding each harvest.
- A climate isotope enrichment factor,  εO 18m−pW,climate, between the water of the grape and the water adsorbed by the plant may be defined. This fractionation depends on climatic/meteorological conditions (air temperature, air humidity, solar radiation and wind) and, most likely, represents the concomitant effect of equilibrium isotope fractionation and nonequilibrium isotope fractionation explained by Gonfiantini [2].
- Solar radiation, wind speed and air temperature have a positive correlation with *δ* ^18^O_m_, while a negative correlation exists between relative humidity and *δ* ^18^O_m_. PCA analysis and correlation coefficients between each individual climate variable and *δ* ^18^O_m_ have been used to explain this behaviour.
- The linear relation between ET_C_ on *δ* ^18^O_m_ has descriptive rather than predictive character.

## Conflicts of Interest

The authors declare no conflicts of interest.

## Funding

No funding was received for this manuscript.

## Supporting information


**Supporting Information** Additional supporting information can be found online in the Supporting Information section. Table S1: Potential evapotranspiration (ET_0_) referred to 2021 year of Oltrepò Pavese sites. Table S2: Water musts *δ* ^18^O values of Oltrepò Pavese sites referred to 2021 harvest. Table S3: Water musts *δ* ^18^O values of Oltrepò Pavese sites referred to 2022 harvest, Table S4: Potential evapotranspiration (ET_0_) referred to 2022 year of Oltrepò Pavese sites. Table S5: Crop evapotranspiration (ET_C_) referred to 2021 year of Oltrepò Pavese sites. Table S6: Crop evapotranspiration (ET_C_) referred to 2022 year of Oltrepò Pavese sites. Table S7: Water musts *δ* ^18^O values and crop evapotranspiration (ETc) of Illasi/Mezzane sites for 2021 harvest years and used in Figure 7. Table S8: Water musts *δ* ^18^O values and crop evapotranspiration (ETc) of Illasi/Mezzane sites for 2021 harvest years and used in Figure 8. Table S9: Solar radiation, wind speed, relative humidity and air temperature values for correlation coefficients related to the Table 3 of the manuscript.

## Data Availability

The data that support the findings of this study are available in the supporting information of this article.
