# Supplementary material for: Factors Influencing the Oxygen Isotopes of Grape Berry Water: Some Remarks
Source: Int J Food Sci. 2026 Jan 5;2026:8946352. doi: 10.1155/ijfo/8946352 (PMC12766274; doi:10.1155/ijfo/8946352)
Supplement: Supplementary file 1 — Supporting Information Additional supporting information can be found online in the Supporting Information section. Table S1: Potential evapotranspiration (ET0) referred to 2021 year of Oltrepò Pavese sites. Table S2: Water musts δ 18O values of Oltrepò Pavese sites referred to 2021 harvest. Table S3: Water musts δ 18O values of Oltrepò Pavese sites referred to 2022 harvest, Table S4: Potential evapotranspiration (ET0) referred to 2022 year of Oltrepò Pavese sites. Table S5: Crop evapotranspiration (ETC) referred to 2021 year of Oltrepò Pavese sites. Table S6: Crop evapotranspiration (ETC) referred to 2022 year of Oltrepò Pavese sites. Table S7: Water musts δ 18O values and crop evapotranspiration (ETc) of Illasi/Mezzane sites for 2021 harvest years and used in Figure 7. Table S8: Water musts δ 18O values and crop evapotranspiration (ETc) of Illasi/Mezzane sites for 2021 harvest years and used in Figure 8. Table S9: Solar radiation, wind speed, relative humidity and air temperature values for correlation coefficients related to the Table 3 of the manuscript. [file IJFO-2026-8946352-s001.docx]

**Supplementary Data**

This file includes potential evapotranspiration ET_0_ (Table S1 and S4), crop evapotranspiration (ET_c_), (Table S5 and S6), δ^18^O water musts values of Oltrepò Pavese (Table S2 and S3), and Illasi-Mezzane (Table S7 and S8) sites for the two different years of study (2021 and 2022). Please note that for Montalto Pavese and Montebello della Battaglia the δ^18^O values are more than one at the same date, because a multisampling was done on the same day. Furthermore, climate parameter for correlation coefficients related to the table 3values are reported in Table S9.

**Table S1** Potential evapotranspiration (ET_0_) referred to 2021 year of Oltrepò Pavese sites

|  | ET_O_ (mm/day) | | | | | |
| --- | --- | --- | --- | --- | --- | --- |
| HARVEST DATE | BORGORATTO MORMOLO | CANEVINO | MONTALTO PAVESE | CIGOGNOLA | MONTEBELLO DELLA BATTAGLIA | SANTA MARIA DELLA VERSA |
| September 07, 2021 | 4.62 | 4.47 | 4.75 | 4.48 | 4.74 | 4.60 |
| September 15, 2021 | 4.57 | 4.29 | 4.71 | 4.41 | 4.66 | 4.54 |
| September 24, 2021 | 4.30 | 4.20 | 4.45 | 4.15 | 4.41 | 4.28 |
| September 30, 2021 | 3.80 | 3.69 | 3.95 | 3.67 | 3.91 | 3.80 |

**Table S2** Water musts δ^18^O values of Oltrepò Pavese sites referred to 2021 harvest

|  | δ^18^O VALUES (‰) | | | | | |
| --- | --- | --- | --- | --- | --- | --- |
| HARVEST DATE | BORGORATTO MORMOLO | CANEVINO | MONTALTO PAVESE | CIGOGNOLA | MONTEBELLO DELLA BATTAGLIA | SANTA MARIA DELLA VERSA |
| September 07, 2021 | 6.73 | 4.93 | 5.40 | 5.58 | 5.79 | 4.74 |
|  |  |  | 6.83 |  |  |  |
|  |  |  |  |  | 6.24 |  |
|  |  |  | 5.99 |  |  |  |
| September 15, 2021 | 5.31 | 4.83 | 5.30 | 6.61 | 7.60 | 6.16 |
|  |  |  | 6.31 |  |  |  |
|  |  |  |  |  | 7.53 |  |
|  |  |  | 6.04 |  |  |  |
| September 24, 2021 | 4.92 | 4.69 | 5.18 | 4.83 | 6.38 | 4.14 |
|  |  |  | 5.97 |  |  |  |
|  |  |  |  |  | 5.65 |  |
|  |  |  | 4.64 |  |  |  |
| September 30, 2021 | 3.12 | 2.39 | 2.94 | 2.40 | 3.88 | 2.23 |
|  |  |  | 3.25 |  |  |  |
|  |  |  |  |  | 3.98 |  |
|  |  |  | 3.37 |  |  |  |

**Table S3** Water musts δ^18^O values of Oltrepò Pavese sites referred to 2022 harvest

|  | δ^18^O VALUES (‰) | | | | | |
| --- | --- | --- | --- | --- | --- | --- |
| HARVEST DATE | BORGORATTO MORMOLO | CANEVINO | MONTALTO PAVESE | CIGOGNOLA | MONTEBELLO DELLA BATTAGLIA | SANTA MARIA DELLA VERSA |
| August 26, 2022 | 6.91 | 7.13 | 7.26 | 6.84 | 7.67 | 5.70 |
|  |  |  | 7.24 |  |  |  |
|  |  |  |  |  | 7.21 |  |
|  |  |  | 7.00 |  |  |  |
| September 05, 2022 | 5.84 | 6.11 | 6.90 | 5.14 | 6.64 | 5.20 |
|  |  |  | 6.84 |  |  |  |
|  |  |  |  |  | 6.09 |  |
|  |  |  | 5.86 |  |  |  |
| September 12, 2022 | 5.43 | 5.09 | 5.32 | 3.86 | 5.84 | 5.12 |
|  |  |  | 5.22 |  |  |  |
|  |  |  |  |  | 5.65 |  |
|  |  |  | 4.97 |  |  |  |
| September 19, 2022 | 3.66 | 3.47 | 4.72 | 3.83 | 4.94 | 4.61 |
|  |  |  | 4.32 |  |  |  |
|  |  |  |  |  | 4.05 |  |
|  |  |  | 3.97 |  |  |  |

**Table S4** Potential evapotranspiration (ET_0_) referred to 2022 year of Oltrepò Pavese sites

|  | ET_O_ (mm/day) | | | | | |
| --- | --- | --- | --- | --- | --- | --- |
| HARVEST DATE | BORGORATTO MORMOLO | CANEVINO | MONTALTO PAVESE | CIGOGNOLA | MONTEBELLO DELLA BATTAGLIA | SANTA MARIA DELLA VERSA |
| August 26, 2022 | 5.93 | 5.78 | 6.17 | 5.71 | 6.16 | 6.18 |
| September 05, 2022 | 5.45 | 5.30 | 5.68 | 5.24 | 5.68 | 5.83 |
| September 12, 2022 | 5.06 | 4.90 | 5.27 | 4.88 | 5.30 | 5.53 |
| September 19, 2022 | 4.64 | 4.47 | 4.83 | 4.47 | 4.86 | 5.19 |

**Table S5** Crop evapotranspiration (ET_c_) referred to 2021 year of Oltrepò Pavese sites

|  | ET_c_ (mm/day) | | | | | |
| --- | --- | --- | --- | --- | --- | --- |
| HARVEST DATE | BORGORATTO MORMOLO | CANEVINO | MONTALTO PAVESE | CIGOGNOLA | MONTEBELLO DELLA BATTAGLIA | SANTA MARIA DELLA VERSA |
| September 07, 2021 | 2.26 | 2.17 | 2.35 | 2.13 | 2.29 | 2.23 |
| September 15, 2021 | 2.25 | 2.18 | 2.36 | 2.12 | 2.29 | 2.22 |
| September 24, 2021 | 2.04 | 1.96 | 2.15 | 1.92 | 2.09 | 2.02 |
| September 30, 2021 | 1.76 | 1.69 | 1.86 | 1.68 | 1.82 | 1.76 |

**Table S6** Crop evapotranspiration (ET_c_) referred to 2022 year of Oltrepò Pavese sites

|  | ET_c_ (mm/day) | | | | | |
| --- | --- | --- | --- | --- | --- | --- |
| HARVEST DATE | BORGORATTO MORMOLO | CANEVINO | MONTALTO PAVESE | CIGOGNOLA | MONTEBELLO DELLA BATTAGLIA | SANTA MARIA DELLA VERSA |
| August 26, 2022 | 2.96 | 2.83 | 3.19 | 2.76 | 3.11 | 3.08 |
| September 05, 2022 | 2.69 | 2.57 | 2.90 | 2.51 | 2.84 | 2.89 |
| September 12, 2022 | 2.47 | 2.35 | 2.66 | 2.31 | 2.62 | 2.73 |
| September 19, 2022 | 2.24 | 2.13 | 2.42 | 2.10 | 2.38 | 2.55 |

**Table S7** Water musts δ^18^O values and crop evapotranspiration (ETc) of Illasi-Mezzane sites for 2021 harvest years and used in Fig. 7

| Harvest date | δ^18^O VALUES (‰) | ETc (mm/day) |
| --- | --- | --- |
| September 02, 2021 | 3.18 | 2.13 |
| September 03, 2021 | 5.38 | 2.12 |
| September 10, 2021 | 5.29 | 2.11 |
| September 11, 2021 | 4.25 | 2.1 |
| September 20, 2021 | 2.37 | 1.62 |
| September 30, 2021 | 2.62 | 1.57 |
| October 02, 2021 | 3.86 | 1.46 |
| October 07, 2021 | 3.85 | 1.95 |

**Table S8** Water musts δ^18^O values and crop evapotranspiration (ETc) of Illasi-Mezzane sites for 2021 harvest years and used in Fig. 8

| Harvest date | δ^18^O VALUES (‰) | ETc (mm/day) |
| --- | --- | --- |
| August 25, 2022 | 5.49 | 2.60 |
| August 26, 2022 | 4.97 | 2.61 |
| August 30, 2022 | 4.98 | 2.56 |
| September 02, 2022 | 3.99 | 2.49 |
| September 05, 2022 | 3.29 | 2.43 |
| September 06, 2022 | 4.34 | 2.42 |
| October 04, 2022 | 3.38 | 1.63 |
| October 18, 2022 | 3.40 | 1.34 |
| October 18, 2022 | 3.82 | 1.34 |

**Table S9** Solar radiation, wind speed, relative humidity, and air temperature values for correlation coefficients related to the table 3 of the manuscript.

| δ^18^O (‰) | Solar radiation $(W\cdot m^{-2})$ | Wind speed $(m\cdot s^{-1})$ | Relative Humidty (%) | Air Temperature (°C) |
| --- | --- | --- | --- | --- |
| 6.91 | 285.02 | 1.88 | 59.90 | 25.97 |
| 5.84 | 262.48 | 1.83 | 62.07 | 25.51 |
| 5.43 | 247.55 | 1.73 | 63.62 | 25.02 |
| 3.66 | 231.33 | 1.66 | 64.73 | 24.09 |
| 7.13 | 285.78 | 1.88 | 55.52 | 25.57 |
| 6.11 | 264.01 | 1.83 | 58.44 | 25.07 |
| 5.09 | 248.46 | 1.73 | 61.20 | 24.64 |
| 3.47 | 232.20 | 1.66 | 62.50 | 23.73 |
| 7.26 | 287.92 | 1.88 | 48.90 | 26.01 |
| 7.24 | 287.92 | 1.88 | 48.90 | 26.01 |
| 7 | 287.92 | 1.88 | 48.90 | 26.01 |
| 6.9 | 266.19 | 1.83 | 51.66 | 25.54 |
| 6.84 | 266.19 | 1.83 | 51.66 | 25.54 |
| 5.86 | 266.19 | 1.83 | 51.66 | 25.54 |
| 5.32 | 251.39 | 1.73 | 53.98 | 25.07 |
| 5.22 | 251.39 | 1.73 | 53.98 | 25.07 |
| 4.97 | 251.39 | 1.73 | 53.98 | 25.07 |
| 4.72 | 234.89 | 1.66 | 55.15 | 24.10 |
| 4.32 | 234.89 | 1.66 | 55.15 | 24.10 |
| 3.97 | 234.89 | 1.66 | 55.15 | 24.10 |
| 6.84 | 289.31 | 1.88 | 67.05 | 25.14 |
| 5.14 | 267.43 | 1.83 | 68.64 | 24.70 |
| 3.86 | 252.99 | 1.73 | 69.67 | 24.25 |
| 3.83 | 236.28 | 1.66 | 70.35 | 23.31 |
| 7.67 | 289.67 | 1.88 | 52.64 | 26.38 |
| 7.21 | 289.67 | 1.88 | 52.64 | 26.38 |
| 6.64 | 268.23 | 1.83 | 55.23 | 25.92 |
| 6.09 | 268.23 | 1.83 | 55.23 | 25.92 |
| 5.84 | 253.85 | 1.73 | 57.07 | 25.47 |
| 5.65 | 253.85 | 1.73 | 57.07 | 25.47 |
| 4.94 | 237.31 | 1.66 | 58.08 | 24.57 |
| 4.05 | 237.31 | 1.66 | 58.08 | 24.57 |
| 5.7 | 288.61 | 1.88 | 65.37 | 25.53 |
| 5.2 | 266.95 | 1.83 | 65.83 | 25.32 |
| 5.12 | 251.94 | 1.73 | 66.04 | 25.07 |
| 4.61 | 235.39 | 1.66 | 65.99 | 24.23 |
| 6.73 | 242.13 | 1.70 | 64.19 | 23.09 |
| 5.31 | 240.27 | 1.70 | 60.71 | 23.00 |
| 4.92 | 216.16 | 1.76 | 61.71 | 21.89 |
| 3.12 | 196.79 | 1.64 | 65.37 | 20.85 |
| 4.93 | 242.46 | 1.70 | 61.21 | 22.91 |
| 4.83 | 241.92 | 1.70 | 54.94 | 22.92 |
| 4.69 | 217.25 | 1.76 | 57.70 | 21.74 |
| 2.39 | 198.25 | 1.64 | 62.70 | 20.67 |
| 5.4 | 245.60 | 1.70 | 58.41 | 23.22 |
| 6.83 | 245.60 | 1.70 | 58.41 | 23.22 |
| 5.99 | 245.60 | 1.70 | 58.41 | 23.22 |
| 5.3 | 244.08 | 1.70 | 53.72 | 23.13 |
| 6.31 | 244.08 | 1.70 | 53.72 | 23.13 |
| 6.04 | 244.08 | 1.70 | 53.72 | 23.13 |
| 5.18 | 220.73 | 1.76 | 55.37 | 22.02 |
| 5.97 | 220.73 | 1.76 | 55.37 | 22.02 |
| 4.64 | 220.73 | 1.76 | 55.37 | 22.02 |
| 2.94 | 199.82 | 1.64 | 58.89 | 20.97 |
| 3.25 | 199.82 | 1.64 | 58.89 | 20.97 |
| 3.37 | 199.82 | 1.64 | 58.89 | 20.97 |
| 5.58 | 247.04 | 1.70 | 70.15 | 22.49 |
| 6.61 | 245.88 | 1.70 | 68.40 | 22.15 |
| 4.83 | 221.03 | 1.76 | 68.96 | 21.10 |
| 2.4 | 201.48 | 1.64 | 71.36 | 20.10 |
| 5.79 | 247.62 | 1.70 | 61.32 | 23.33 |
| 6.24 | 247.62 | 1.70 | 61.32 | 23.33 |
| 7.6 | 244.86 | 1.70 | 57.08 | 23.19 |
| 7.53 | 244.86 | 1.70 | 57.08 | 23.19 |
| 6.38 | 221.28 | 1.76 | 58.35 | 22.14 |
| 5.65 | 221.28 | 1.76 | 58.35 | 22.14 |
| 3.88 | 199.57 | 1.64 | 61.85 | 21.17 |
| 3.98 | 199.57 | 1.64 | 61.85 | 21.17 |
| 4.74 | 245.35 | 1.70 | 68.69 | 22.05 |
| 6.16 | 244.21 | 1.70 | 66.92 | 21.64 |
| 4.14 | 219.57 | 1.76 | 67.59 | 20.49 |
| 2.23 | 200.18 | 1.64 | 69.67 | 19.48 |
